# Supplementary material for: Anti-Arthritogenic Property of Interleukin 10-Expressing Human Amniotic MSCs Generated by Gene Editing in Collagen-Induced Arthritis
Source: Int J Mol Sci. 2022 Jul 18;23(14):7913. doi: 10.3390/ijms23147913 (PMC9320257; doi:10.3390/ijms23147913)
Supplement: Supplementary file 1 [file ijms-23-07913-s001.zip › ijms-1767204-supplementary.pdf]

Supplementary Data

Supplementary materials and methods

Cell proliferation assays

Mouse CD3+ splenocytes were isolated using MACS (Miltenyi Biotec, Bergisch Gladbach, Germany) and plated in triplicate in 96-well plates at 2 × 10^3 cells per well. The cells were starved with 0.1 % FBS for 24 h and then treated with three kinds of culture medium, AMM, AMM/I, or 10 % serum (control). After treatment, the cell proliferation was measured using a CCK-8 assay kit (Dojindo, Japan). The CCK-8 assay kit was treated with DMEM, and then 100 μl was added to each well and they were incubated for 18 h at 37 °C. The absorbance was measured at 450 nm with a spectrometer.

Supplementary Figure Legend

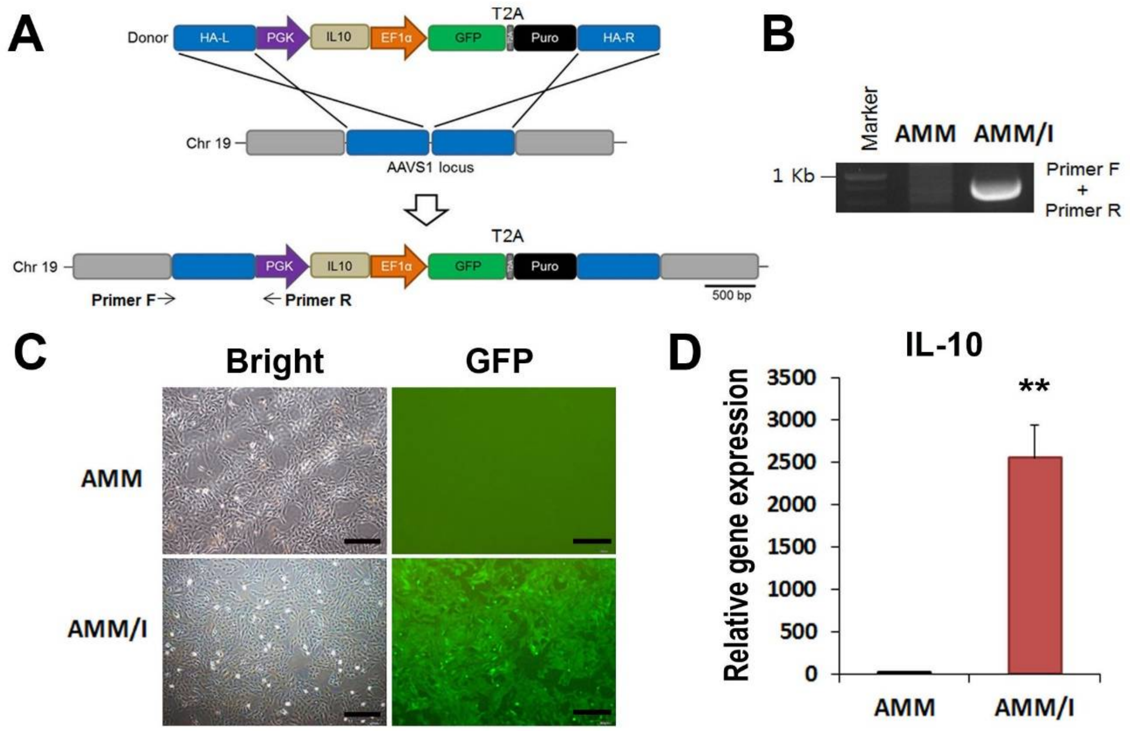

Supplementary Figure S1. The generation of an AMM/I cell line using TALEN gene editing. (**A**) A schematic diagram of the donor vector carrying IL-10 donor plasmid DNA. The expression cassette containing the PGK promoter-driven IL-10 and EF1α promoter-driven GFP-T2A-puromycin was inserted into the AAVS1 site via homology-directed repair (HR). The locations of the primers for junction detection are indicated (primers F and R). Abbreviations: HA-L, left homology arm; HA-R, right homology arm; PGK, phosphoglycerate kinase promoter; EF1α, elongation factor-1 alpha promoter; Puro, puromycin. (**B**) The success of the insertion of the donor plasmid was confirmed in the control AMM and AMM/I using a junction PCR technique. (**C**) A GFP expressing AMM/I. The transfected cells were selected based on puromycin, followed by FACS sorting. Bars = 500 μm. (**D**) The expression levels of IL-10 were examined using qRT-PCR. **$p < 0.01$, $n = 4$.

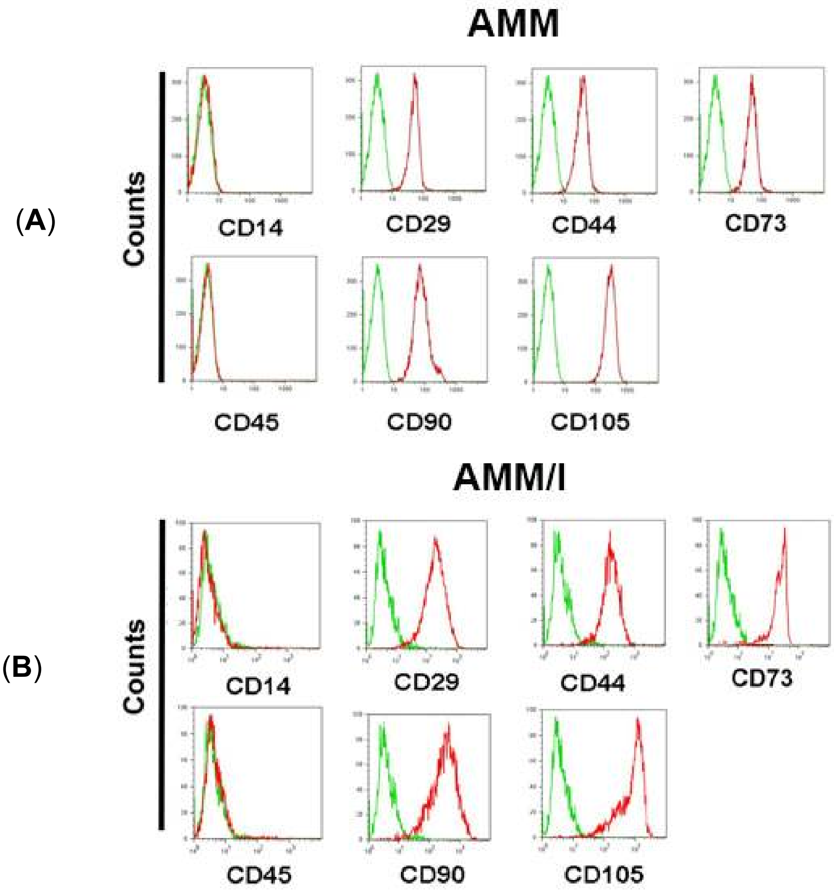

Supplementary Figure S2. The characteristics of AMM/I. (**A**) A microscopic view of AMM/I (passage 3) (bar: 200μm). (**B**) Representative FACS surface markers of the AMM exhibiting MSC-specific cell surface markers. A FACS analysis showed that the AMM minimally expressed hematopoietic cell markers (CD14 and CD45) and MHC class II molecules (HLA-DR), but expressed high levels of the MSC-specific markers CD29, CD44, CD73, CD90, and CD105. The green color represents the isotype control, and the red color represents the specific monoclonal antibody.

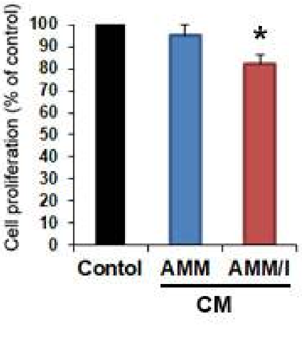

Supplementary Figure S3. T cell proliferation assay. The effects of AMM/I on CD3+ T cells

proliferation are shown. T cells treated with 10 % fetal bovine serum (FBS) were used as the control. Cell counts were conducted after one day of culture. *$p < 0.05$, $n = 5$ per group.
